# Video Conferences through the Internet: How to Survive in a Hostile Environment

**DOI:** 10.1155/2014/860170

**Published:** 2014-01-28

**Authors:** Carlos Fernández, Jose Saldana, Julián Fernández-Navajas, Luis Sequeira, Luis Casadesus

**Affiliations:** Communication Technologies Group (GTC), Aragon Institute of Engineering Research (I3A) EINA, University of Zaragoza, 50018 Zaragoza, Spain

## Abstract

This paper analyzes and compares two different video conference solutions, widely used in corporate and home environments, with a special focus on the mechanisms used for adapting the traffic to the network status. The results show how these mechanisms are able to provide a good quality in the hostile environment of the public Internet, a best effort network without delay or delivery guarantees. Both solutions are evaluated in a laboratory, where different network impairments (bandwidth limit, delay, and packet loss) are set, in both the uplink and the downlink, and the reaction of the applications is measured. The tests show how these solutions modify their packet size and interpacket time, in order to increase or reduce the sent data. One of the solutions also uses a scalable video codec, able to adapt the traffic to the network status and to the end devices.

## 1. Introduction

The commercial success of portable electronic devices, in conjunction with the ubiquitous presence of the Internet, is changing our communications habits. In addition, user's mobility is higher, and this fact increases our need for means of communication. In this environment, new alternatives to traditional telephony are becoming more and more popular. One example is the migration from voice to data connections, which are being widely used for instant messaging or voice over IP (VoIP). Another significant example is the popularization of video conference solutions, which are no longer restricted to corporate environments but have also become popular on our daily communications.

In home environments, video conference presents a lot of clear advantages with respect to a simple phone call: first, the possibility of viewing the other party provides a greater sense of closeness; second, it does not increase your phone bill at the end of the month. At the same time, in corporate environments, video conferences provide a better communication with other offices, displaced staff, or even remote clients, thus saving economic and environmental journey associated costs [1].

As a consequence of these factors, there is a wide variety of video conference solutions, ranging from dedicated devices, developed by the leading telecom equipment vendors, to software-based solutions, whose only requirement is to install an application.

The problem that all these solutions must face is to provide an acceptable quality of service (QoS) when establishing a video conference using the Internet, a *best effort *network which lacks delay guarantees. The public Internet presents a certain degree of unpredictability, since its network conditions may vary as a consequence of different factors [2]: traffic fluctuations and network congestion may change end-to-end delay or may cause delay variation (jitter), bandwidth reduction, or packet loss bursts, which directly affects the QoS of the running communications.

In addition, each of the access technologies presents its own particular issues, which have to be taken into account. For example, DSL presents a strong asymmetry, with a very low bandwidth in the uplink. This is not optimal for video conference, which also requires uploading a video stream. To make things worse, many end devices use wireless connections in the last hop, which may add delay and jitter to the packets.

The present paper analyzes some of the techniques that video conference solutions use in order to provide a good QoS in the hostile environment of the public packet switched networks. The mechanisms they use so as to adapt their traffic according to the network status are studied.

Two different solutions are compared, which constitute significant examples of video conference systems with different schemes and designs: first, *Vidyo*, a corporate solution that uses an adaptive architecture based on video layers, using a scalable video codec. It is able to dynamically adapt the video stream, according to the conditions of the network and the technical characteristics of the end terminal playing the video. The second solution tested is *Skype*, a video conference solution which has become very popular and has strongly contributed to the popularization of real-time communications over the Internet.

These two solutions rely on very different architectures and mechanisms, so the comparison between them will be illustrative of what is needed in order to make things work in the hostile environment of public packet switching networks.

The rest of the paper is organized as follows: in Section 2, a succinct review of the video conference systems and their architectures is presented; Section 3 will present the environment in which the tests with the two solutions have been deployed; the obtained results will be presented in Section 4; the paper ends with a summary of the conclusions.

## 2. Video Conference Solutions

### 2.1. Using the Internet for Videoconferencing

The first attempts for creating video conference systems using traditional telephony did not succeed due to the bandwidth scarceness, the inefficient video compressing techniques, and the high costs. As an example, AT&T commercialized the *Picturephone *[3], but the sales were really low. With the arrival of ISDN, with a bandwidth of 128 kbps, the possibility of setting up a video conference became a reality. One of the first proposals was developed by *PictureTel*. However, these solutions required specific devices, so they were only developed for corporate or medical environments.

But it was in the 90s when videoconferencing through the Internet became popular, although it was widely spread out later with the rising of desktop applications, as *Skype.* This was possible thanks to the improvement in video compression techniques. From 2005 high resolution systems have also been developed [4]. Currently, high resolution video conferences are becoming feasible not only for desktop devices, but also for portable devices using wireless networks.

### 2.2. Models for Providing the Service

The two basic models for a video conference system are peer-to-peer (P2P) and client-server. In P2P systems, all the nodes can act as a client or as a server for the rest of the nodes. This model is frequently used in audio and video streaming systems [5]. Although some basic functions (authentication, accounting, etc.) are centralized, the nodes can self-organize when a multi conference is set, thus increasing the scalability, improving the system's resilience, and having a better distribution of the processing costs between the nodes. Another advantage is that they present a lower latency, since the traffic does not have to go through the server, but it is directly exchanged between peers when possible.

One of the main examples of P2P architecture nowadays, which has been largely studied in the scientific literature, is *Skype *[6]. In [7] two kinds of nodes were defined: supernodes, those who have a public IP, and the rest. Supernodes constitute a P2P network between them, and they are in charge of retransmitting the traffic to the normal nodes, which are usually behind a NAT.

In client-server (or centralized) architectures, there is a central node with a high processing capacity and bandwidth, which permits reducing the requirements of the clients. It should be taken into account that these systems are expected to work in portable devices as smart phones or tablets, with a limited processing capacity, bandwidth, and energy. The system management is easier due to the centralized scheme, and scalability is achieved by the addition of new components to the central server. Redundancy is recommended in order to increase the system's resilience. In the case of *Vidyo, *the central node is called *Vidyorouter*, able to set up the conferences between different nodes, be it a video conference room, a desktop, a laptop, or a mobile device. In order to participate, the end user has only to download and install an application on the device. The central router is able to combine the video streams received from each device, adapting them according to the network conditions and to the characteristics of the end device. For that aim, it leverages on a scalable video codec.

### 2.3. Network Adaptation Mechanisms

As said in the introduction, setting up a video conference through the Internet constitutes a challenge, due to the lack of a delay limit and the variability of the impairments that affect the traffic. It should be remarked that users are accustomed to the reliability of traditional telephony, so they are not very tolerant to drops or quality degradation during the call. Thus, this section first summarizes the mechanisms used by video conference systems in order to work properly in this environment.

Scalable video codecs were designed with the aim of adapting the quality of a stream when bandwidth is not guaranteed. H.264/SVC is a scalability extension added to the H.264/AVC codec [8]. These two codecs were standardized by the *Joint Video Team *from ITU-T VCEG and ISO/IEC MPEG.

Scalable codecs rely on three kinds of scalability: temporal, which is able to tune the number of frames per second; spatial, which is able to modify the image size; and finally the image quality which can also be adapted, with different values of signal-noise relationship. A layer is defined as a combination of these characteristics. First, a “base layer” is defined, which must always be transmitted, and provides a basic quality. In addition, a number of “enhancement layers,” only transmitted when possible, are able to improve the video. They can only be decoded in junction with the base layer. Thus, a video using a scalable codec can be transmitted with different quality levels, just by sending different numbers of layers, without the need of recoding it. This is especially interesting for video conferencing, since it allows a degree of flexibility when the video has to be adapted according to the network status or due to the characteristics of the end device.

In addition to the use of robust and scalable codecs, video conference solutions use different mechanisms to react against network changes. In the literature, some studies have explored this topic, mainly focused on *Skype*: in [9, 10] this application was studied, taking into account that the source code is not available. Some of the adaptation mechanisms identified and characterized were as follows.

- In the transport layer, *Skype *uses UDP, but it is able to switch to TCP if the firewall or NAT policies do not permit UDP traffic.
- In the first 20 seconds of a call, *Skype* adds redundancy to its traffic, in order to determinate the network status, sending alternatively big and small packets. After that interval, if network conditions are good enough, it stops sending big packets. As a result, bandwidth is significantly higher during the first 20 seconds of a conversation.
- Different techniques are used according to the different network impairments: when packet loss is detected, some redundant information is transmitted, so packet size increases. If bandwidth gets scarce, the transmission rate is reduced, thus augmenting interpacket time.

All these mechanisms allow these services to work properly under very different conditions, and they are one of the main causes of the commercial success of these solutions in the last years.

## 3. Tests Setup

**Figure 1:**
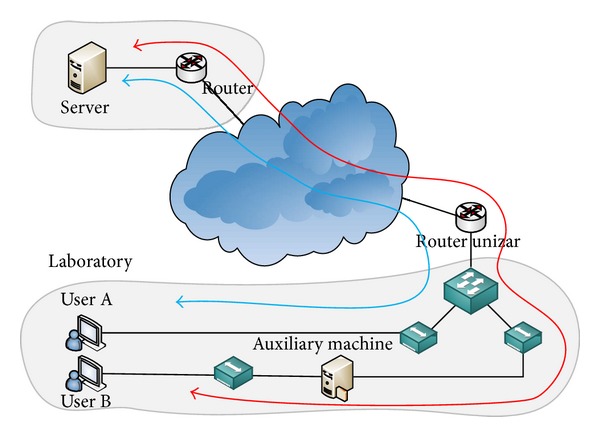
Test scenario.

The test scenario illustrated in Figure 1 has been used in order to reproduce the real environments in which video conference solutions are used.

Two computers creating the video conference have been included in the lab in University of Zaragoza (unizar). In the case of *Vidyo, *the video conference server *(Vidyorouter) *is located in a remote town (Huesca, 100 km away). In the case of *Skype, *the communication is set between the two computers, using the *Skype *public network through the Internet.

A number of hubs, which allow us to capture the traffic, have also been included.

One of the users' computers includes a camera (resolution 800 × 450), which is pointed to a screen playing a high-movement video (the continuously played video is “football,” typically used in research articles as an example of fast movement (http://media.xiph.org/video/derf/)).

An auxiliary machine has also been included, being in charge of adding different network impairments in the uplink and in the downlink. A Proxy ARP is used in order to make all the traffic pass through it. The limitations are added by means of Linux *tc *(traffic control) (http://tldp.org/HOWTO/Traffic-Control-HOWTO/). Bandwidth is limited by the use of a token bucket Filter with a certain rate. The buffer size has been set to 104 Kbytes, and the *burst *parameter to 2 Kbytes. Packet loss and network delay are added by means of Linux *Netem* (http://www.linuxfoundation.org/collaborate/workgroups/networking/netem).

As a preliminary test, in order to avoid unexpected network limitations, the available bandwidth between the two networks was measured using IPERF (https://code.google.com/p/iperf/). The reported bandwidth is roughly 90 Mbps, which is high enough, taking into account that video conferences may require up to 2 Mbps.

The computers are commodity machines using Intel Core i3 CPUs. The user's PCs run Windows 7, and the auxiliary machine runs Linux Debian 2.6.32. The transmission and reception bandwidth are set to *auto *in both applications during the tests, so the network is the only factor limiting the throughput.

## 4. Tests and Results

This section presents the results, mainly in terms of the bandwidth generated by the video conference solution, and it also details interpacket time and packet size, in order to get a clearer idea of the mechanisms employed by the applications. It must be taken into account that we do not have access to the source code of the applications, so we will have to guess the causes of the different mechanisms modifying the traffic, as done in previous research [6, 7, 9, 10].

Bandwidth limitations, different rates of packet loss, and artificial delays will be first added in the downlink. Afterwards, the different behavior when the limitations appear in the uplink will specifically be explained when required.

### 4.1. Bandwidth Limitations (Downlink)

#### 4.1.1. *Vidyo*

**Figure 2:**
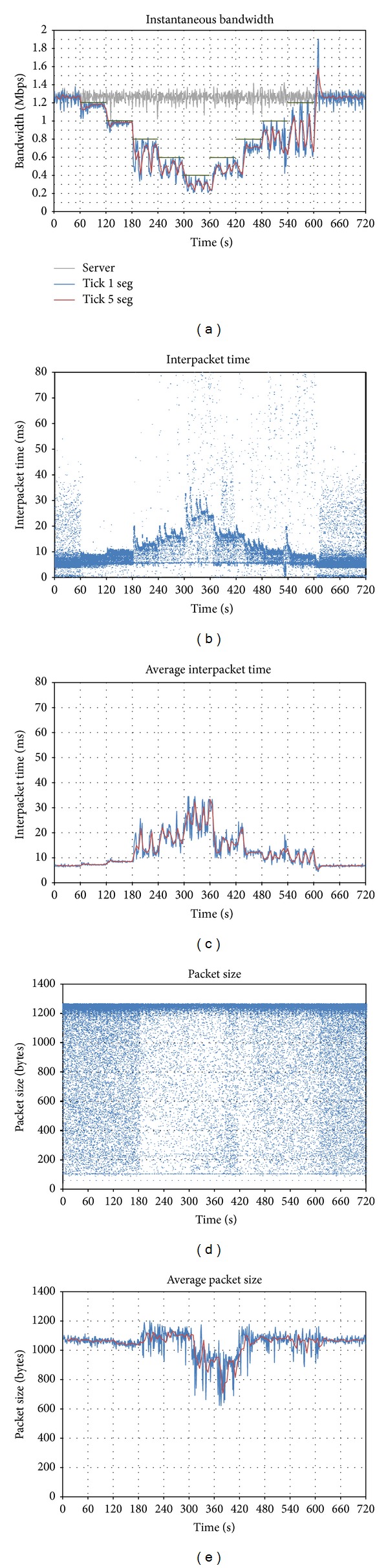
Effect of downlink bandwidth limitations on *Vidyo*: (a) evolution of sent throughput; (b) interpacket time; (c) average interpacket time; (d) packet size; (e) average packet size.

A video conference of 720 seconds is established, adding different bandwidth limitations in the downlink, as shown in the green lines of Figure 2(a). Every 60 seconds, the bandwidth is reduced in 200 kbps. From *t* = 360, the bandwidth is augmented, until the limitation is totally removed at *t* = 600.

The grey graph represents the bandwidth amount sent by the generator client and received by the server; the blue one is the bandwidth sent by the server to the destination client, averaged every 1 sec (tick = 1 s); the red line represents the traffic sent to the client, averaged every 5 seconds (tick = 5 s). All the video packets are UDP.

The first thing that can be observed is that the application is able to adapt its traffic very fast, according to the available bandwidth. For that aim, it uses an oscillation mechanism, which alternatively increases and decreases its throughput.

It can also be observed that the grey line remains constant during the whole conference, so another conclusion can be drawn: the client that generates the video does not reduce the quality, even though the *Vidyo *server is sending a lower quality stream to the destination. The reason is that there could be other clients in the conference, so for the server it is better to get a high quality stream and to adapt it to each client, by the use of the scalable codec.

At the beginning, while there is no bandwidth limitation, *Vidyo *generates 1.3 Mbps, with an average interpacket time (Figures 2(b) and 2(c)) of 7 ms (roughly 150 packets per second). The size of the packets is below 1,100 bytes, although it has a wide range of variation (Figures 2(d) and 2(e)).

When bandwidth limitation is first set (*t* = 60), the traffic sent by the server is reduced. But if the bandwidth limitation becomes more severe (*t* = 180), a different behavior can be observed: bandwidth oscillations appear, presenting a period between 16 and 20 seconds. It can be observed that the throughput oscillates between the bandwidth limit and half the limit: from *t* = 180 to *t* = 240 it can clearly be seen that the generated bandwidth varies from 0.4 to 0.8 Mbps.

Finally, when the limitation is removed, a traffic peak appears. The cause for this can be that the application is trying to estimate the available bandwidth. For that aim, it keeps on increasing the bandwidth, until a certain point in which it confirms that there is no limitation, so it switches to the status it had at the beginning.

#### 4.1.2. *Skype*

**Figure 3:**
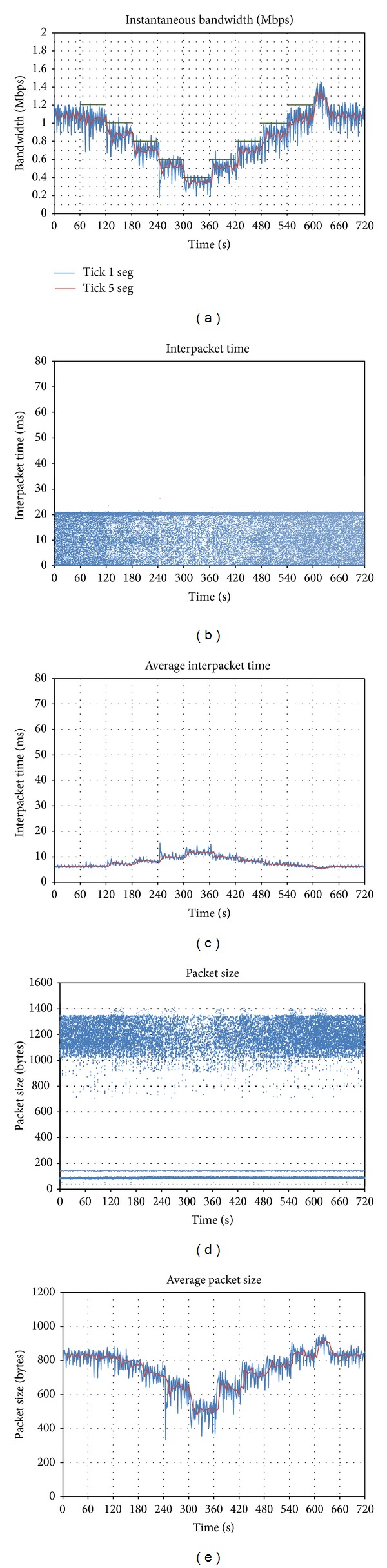
Effect of downlink bandwidth limitations on *Skype*: (a) evolution of sent throughput; (b) interpacket time; (c) average interpacket time; (d) packet size; (e) average packet size.

The same test has been repeated with *Skype, *and the results are shown in Figure 3. In this case, there is no grey line, since the client generator is the one that adapts the bandwidth, instead of letting the server do it. We should remember that *Skype *is a peer-to-peer solution, so traffic adaptation is deployed by the peers and not by a server.

It can be seen that *Skype *is also able to adapt its traffic to the network status, and in this case a single behavior is observed: the range of oscillation is always the same despite the bandwidth limitation. In *t* = 600, a peak similar to the one produced by *Vidyo *appears, with the aim of exploring the status of the network.

With regard to interpacket time (Figures 3(b) and 3(c)), it is roughly constant during the whole test, so we can conclude that bandwidth adaptation is mainly based on the modification of packet size (Figures 3(d) and 3(e)). Regarding packet size distribution, different fringes are observed: UDP video packets are between 1,000 and 1,400 bytes, and smaller audio and synchronization packets are below 200 bytes.

### 4.2. Packet Loss (Downlink)

This section will explore the adaptation mechanisms of these video conference solutions when packet loss in the downlink appears. For that aim, the auxiliary machine is configured in order to add an increasing value of packet loss every 60 seconds, from 0 to 10%, using steps of 1%.

#### 4.2.1. *Vidyo*

**Figure 4:**
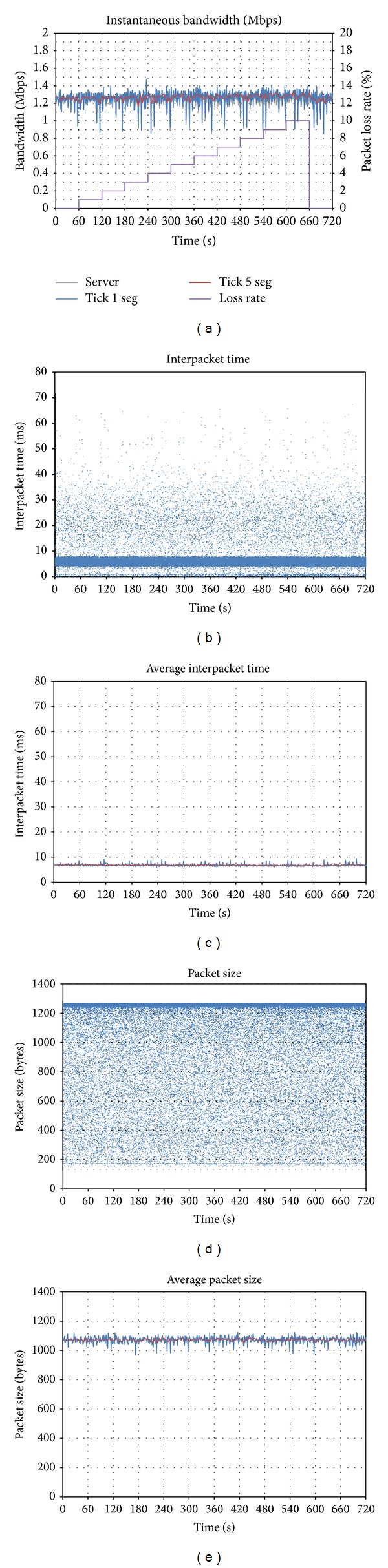
Effect of downlink packet loss on *Vidyo*: (a) evolution of sent throughput; (b) interpacket time; (c) average interpacket time; (d) packet size; (e) average packet size.

Figure 4(a) shows the evolution of the generated bandwidth. The packet loss rate is represented by the purple line. It can be observed that the instantaneous bandwidth does not change with packet loss. Only a slight increasing tendency can be appreciated from *t* = 60 to *t* = 600, which may correspond to some signaling flow, which requires retransmission in case of packet loss.

This lack of reaction is confirmed in Figures 4(b)–4(e), where no evolution can be appreciated in packet size or interpacket time, which remain the same despite the packet loss rate. In order to understand this behavior, we should remember that *Vidyo *relies on a strong coding technology, which is able to perform well even in the presence of packet loss, so the application sends the stream normally.

#### 4.2.2. *Skype*

**Figure 5:**
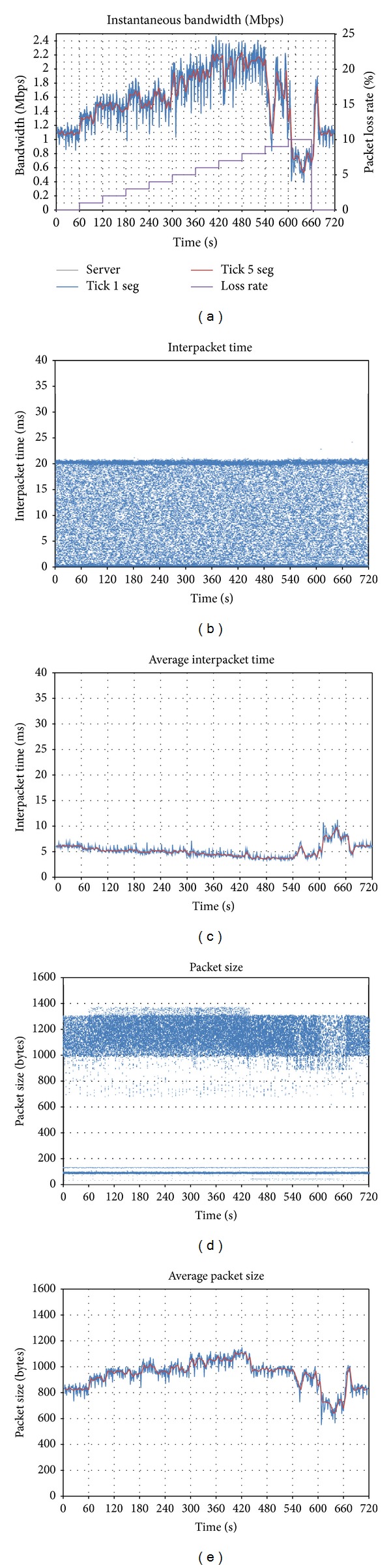
Effect of downlink packet loss on *Skype*: (a) evolution of sent throughput; (b) interpacket time; (c) average interpacket time; (d) packet size; (e) average packet size.

In contrast with the previous behavior, Figure 5 shows that *Skype *does react to packet loss. The bandwidth generated by the client is significantly modified when packet loss is detected. As soon as packet loss appears (*t* = 60), *Skype *reacts increasing the traffic sent from the initial 1.1 Mbps to 1.3 Mbps. This increase becomes more and more significant as the packet loss rate grows: traffic is above 2 Mbps when packet loss is 9%.

As reported in [9], *Skype* implements different mechanisms so as to react to network conditions. If packet loss is detected, it adds redundant data blocks to the messages. This fits the present results reporting that packet size gets increased (Figures 5(d) and 5(e)). Packets of 1,400 bytes are sent from *t* = 60 to *t* = 420, when packet loss is under 7%. Packets become smaller at that moment, and interpacket time is reduced (Figures 5(b) and 5(c)).

Another change of the behavior can be observed in *t* = 540, when packet loss reaches 9%: the generated bandwidth is then reduced to 1 Mbps. *Skype *decides not to keep on increasing its traffic, estimating that packet loss is too high for the videoconference. This behavior is stressed when packet loss becomes 10% (*t* = 600): the bandwidth falls to 0.8 Mbps.

We can conclude this section saying that *Skype *implements different mechanisms to react against packet loss: it reduces the interpacket time, and it also adds redundancy. However, when a certain threshold is reached, a different behavior is observed in which it reduces the generated bandwidth.

### 4.3. Additional Delay (Downlink)

The question we will try to answer in this section is as follows: do the applications include any mechanism to compensate network delay? With that purpose, different delays (orange line) are added, from 50 to 300 ms, beginning in *t* = 60. Finally, in *t* = 420 the additional delay is removed.

#### 4.3.1. *Vidyo*

**Figure 6:**
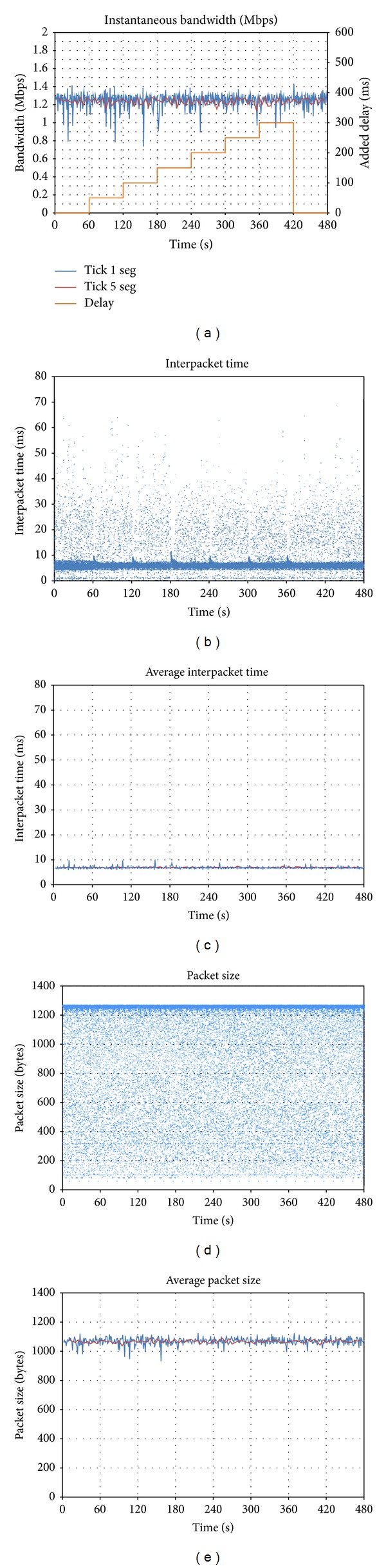
Effect of delay on *Vidyo*: (a) evolution of sent throughput; (b) interpacket time; (c) average interpacket time; (d) packet size; (e) average packet size.

In the case of *Vidyo*, Figure 6(a) does not show any significant modification of the generated bandwidth. However, a peak and a subsequent reduction can be appreciated in the interpacket time graph (Figure 6(b)). A possible explanation of this behavior is that the application interprets it as a transient network congestion status. After 10 seconds, it notices that the delay is not reduced, so it returns to its stationary behavior. The packet size is not modified during the test (Figure 6(c)).

#### 4.3.2. *Skype*

**Figure 7:**
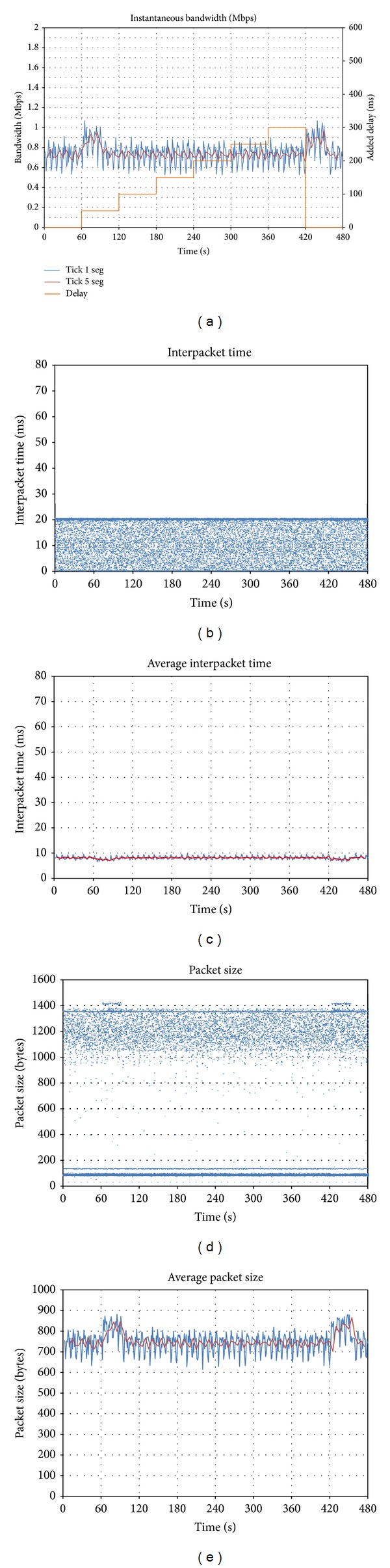
Effect of delay on *Skype*: (a) evolution of sent throughput; (b) interpacket time; (c) average interpacket time; (d) packet size; (e) average packet size.

*Skype *also reacts to delay. It can be observed that in *t* = 60, when the additional delay appears for the first time, the application is able to detect this network impairment, and it then acts in a similar way to the case of packet loss; that is, it augments the generated bandwidth (Figure 7(a)), by generating bigger packets (Figure 7(d)), similar to what could be observed in Figure 5(d)). Interpacket time is not modified (Figures 7(b) and 7(c)). However, after 30 seconds, it notices that the delay increase was not transient, so it returns to its normal behavior.

Something similar happens in *t* = 420: when additional delay is removed, *Skype *detects a modification in network conditions, and it sends a higher amount of traffic in order to test the new status.

The results of [9] reported that *Skype *did not react against delay. In the presented tests, it could be observed that it tries to react, but in the end there is nothing it can do to compensate a constant delay, caused by propagation in the network.

### 4.4. Limitations in the Uplink (*Vidyo*)

This section summarizes the results obtained when the limitations are in the uplink. These new uplink tests only make sense in the case of *Vidyo, *since it has a central server, and in some cases the behavior may present some differences with respect to the downlink limitations. In the case of *Skype, *the obtained results are the same, since *Skype* is a peer-to-peer solution, so it is affected in a similar way by uplink or downlink limitations.

**Figure 8:**
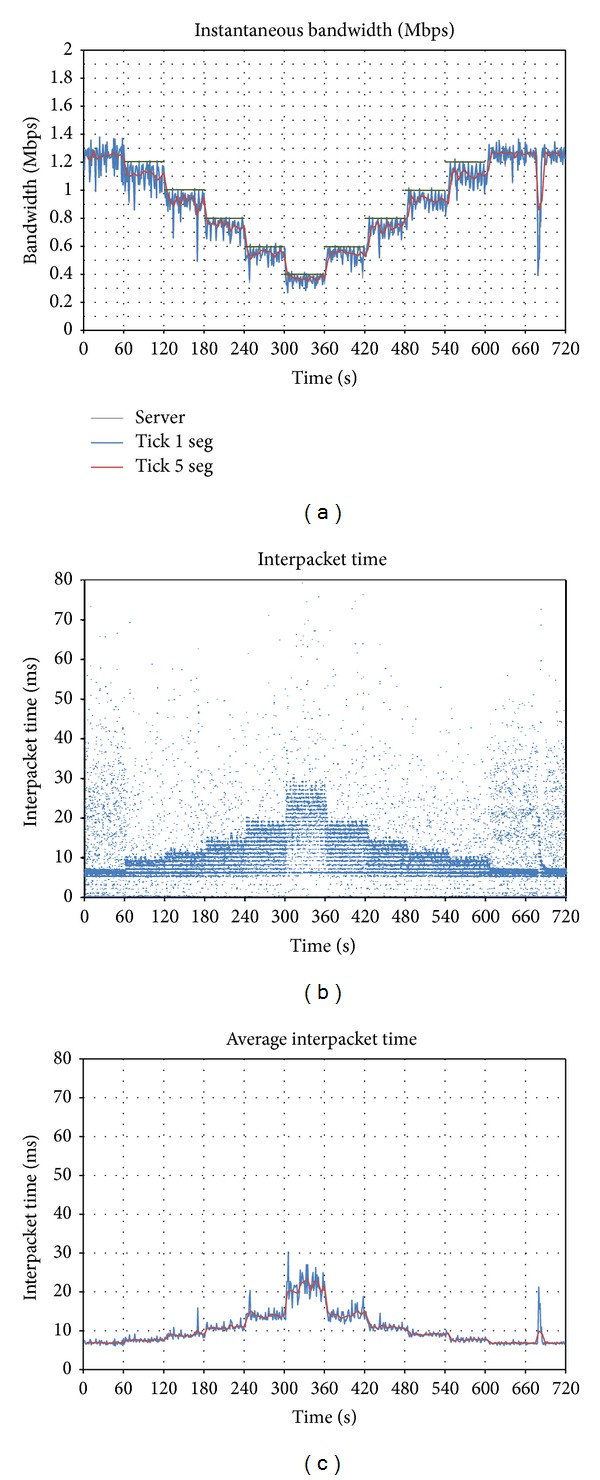
Effect of uplink bandwidth limitations on *Vidyo*: (a) evolution of sent throughput; (b) interpacket time; (c) average interpacket time.

First, a test limiting the bandwidth of *Vidyo *has been deployed (Figure 8(a)). A first difference with respect to Figure 2(a) can be observed: in this case, the grey line (traffic arriving to the server) cannot be observed, since it is behind the blue one (traffic sent to the receiver). The cause is that the limitations in the uplink make the client generate a bandwidth-limited stream, and the server only retransmits it to the receiver, since it has bandwidth enough in the downlink. Thus, the value of the throughput is the same in both cases.

But there is an additional difference: the size of the bandwidth oscillations in this case remains the same during the whole test, whereas two different cases could be distinguished (small and high oscillations) when the limitation was in the downlink.

As a final remark, the traffic peak when limitations are removed (*t* = 600) cannot be observed, so the system has in this case a less aggressive behavior. The peak in *t* = 680 was caused by a transient failure in the network.

Regarding interpacket time (Figures 8(b) and 8(c)), it can be observed that the points tend to be grouped in stripes, with a distance of 1 ms between them. This reveals that the application is using a clock with this period in order to send the packets. This could not be observed in the downlink scenario (Figure 2(b)), since the capture was done after the packets had traversed the network, which adds a certain amount of jitter, so this phenomenon cannot be appreciated.

The graphs corresponding to packet loss and delay are not presented here, since the behavior is the same observed when the limitations are in the downlink.

### 4.5. Comparison of the Responsiveness Uplink-Downlink (*Vidyo*)

**Figure 9:**
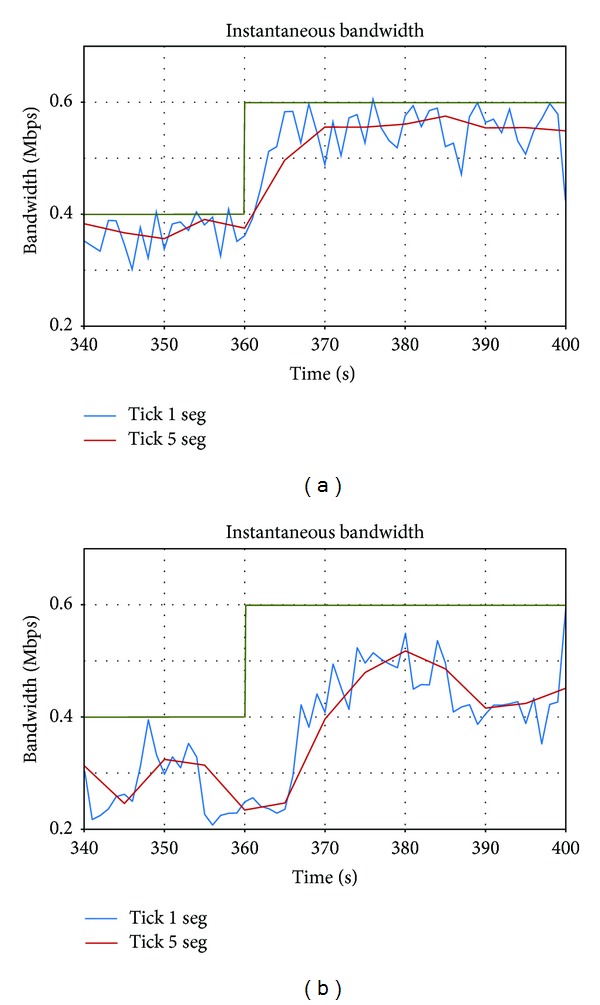
Instantaneous bandwidth of *Vidyo* with (a) bandwidth limitations in the uplink; (b) bandwidth limitations in the downlink.

In this section the responsiveness of the application in both cases is compared: when limitations appear in the uplink and in the downlink.  Figure 9(a) presents a zoom of Figure 8(a) (uplink), and Figure 9(b) presents a zoom of Figure 2(a) (downlink), between seconds 340 and 400, when the available bandwidth is augmented from 0.4 to 0.6 Mbps (*t* = 360).

When the limitations appear in the uplink, the adaptation is faster: the application increases its bandwidth just after *t* = 360. However, if the limitations appear in the downlink, the system reacts after six seconds. This different behavior can be explained according to the different period of the bandwidth oscillations, which is shorter when the limitations are in the uplink.

## 5. Conclusions

This paper has explored the mechanisms used by video conference solutions in order to react when network conditions are modified. For that aim, two different solutions, one with a client-server architecture and another one using a peer-to-peer scheme, have been selected. A test environment has been created, including an auxiliary machine able to artificially add bandwidth limitations, packet loss, and network delay.

Different tests have been run, reducing and augmenting the network impairments, in order to observe the reaction of the applications, in terms of generated bandwidth, interpacket time, and packet size.

The results show that both solutions dynamically react according to the network status and are able to detect the variations very fast. In some cases both applications use similar mechanisms, but in others they rely on different solutions. As an example, one of the solutions reacts against packet loss with an increase of redundancy, whereas the other one does not modify its traffic, trusting the robustness of its scalable video codec. All these tests will be useful so as to know if the mechanisms of the video conference applications are coherent with the network features that can be found in each particular case.
